# Characterization of Aging-Related Genes to Predict Prognosis and Evaluate the Tumor Immune Microenvironment in Malignant Melanoma

**DOI:** 10.1155/2022/1271378

**Published:** 2022-03-24

**Authors:** Ni Zeng, Chenrui Guo, Yajun Wang, Lin Li, Xi Chen, Shaoying Gao, Feng Jiang, Bilan Cao

**Affiliations:** ^1^Department of Dermatology, Affiliated Hospital of Zunyi Medical University, Zunyi 563003, China; ^2^Department of Abdominal Oncology, The Second Affiliated Hospital of Zunyi Medical University, Zunyi 563003, China; ^3^Department of Endocrinology, Taizhou Clinical Medical School of Nanjing Medical University (Taizhou People's Hospital), Taizhou, Jiangsu, China; ^4^Department of Plastic Surgery, Affiliated Hospital of Zunyi Medical University, Zunyi 563003, China; ^5^Department of Neonatology, Obstetrics and Gynecology Hospital of Fudan University, No. 419, Fangxie Road, Shanghai 200011, China

## Abstract

**Objective:**

Malignant melanoma (MM) is one of the most malignant types of skin cancer and its incidence and mortality rates are increasing worldwide. Aging is well recognized as a significant risk factor for cancer. However, few studies have analyzed in depth the association between aging-related genes (AGs) and malignant melanoma prognosis with tumor immune microenvironment.

**Methods:**

Here, we downloaded 471 MM patients from The Cancer Genome Atlas (TCGA) with RNA sequence and clinicopathological data. 58 AGs from the TCGA dataset were examined using Cox regression and the LASSO assay. As a result, a gene signature for aging-related genes was created. The time-dependent ROC curve and Kaplan–Meier analysis were calculated to determine its predictive capability. Moreover, we created a nomogram for the clinicopathologic variables and the AGs gene signature to determine overall survival (OS). We also explored the association between three immune checkpoints, immune cell infiltration, and the aging-related gene signature.

**Results:**

We established an aging risk model to identify and predict the immune microenvironment in malignant melanoma. Then we developed and validated a prognosis risk model using three AGs (CSNK1E, C1QA, and SOD-2) in the GSE65904 dataset. The aging signature was positively associated with clinical and molecular characteristics and can be used as a prognostic factor for malignant melanoma. The low aging risk score was associated with a poor prognosis and indicated an immunosuppressive microenvironment.

**Conclusions:**

To summarize, we established and validated a model of aging risk based on three aging-related genes that acted as an independent prognostic predictor of overall survival. Besides, it also characterized the immune response in the malignant melanoma microenvironment and could provide a potential indicator of individualized immunotherapy in malignant melanoma.

## 1. Introduction

Malignant melanoma is the most common type of malignant transformation of melanocytes. It is also the most destructive type of skin cancer, responsible for nearly 60,700 deaths worldwide per year, making it the leading cause of skin cancer-related deaths [1]. Although surgical resection of the primary tumor is a promising therapeutic option for the majority of limited-stage melanomas, treatment options for extensive-stage melanomas are more difficult, since the majority of single or even combination therapies are only successful in a small percentage of patients [2]. Despite promising clinical outcomes, the prognosis for advanced cases is still poor, with a 5-year survival rate of just over 20% [3]. As a result, effective and precise biomarkers are needed for early diagnosis and tailored intervention strategies to reduce the mortality risk of patients with MM.

Cancer is a disease of aging [4]. Aging is characterized as a gradual deterioration in internal physiological function over time and as a potential risk indicator for several chronic diseases, such as a tumor, which has recently become a popular issue in cancer research [5, 6]. Senescence cells play a vital role in the aging process and the growth of cancers [7]. Senescent cells have a highly complex effect on tumors and can be both beneficial and detrimental. Senescent neoplastic cells caused by oncogenesis can initiate cell cycle arrest, which appears to be a potentially effective antitumor mechanism [8]. Senescent cells, on the other hand, may have the opposite effect on nearby cancer cells and are intimately linked to the secretion of senescence-associated secretory phenotype (SASP) factors [4, 9]. AGs are involved in the control of cellular senescence, which not only inhibits tumors but also promotes their growth, invasion, metastasis, proliferation, and poor prognosis [10]. However, few studies have conducted a systematic examination of the relationship between AGs and MM prognosis. Additionally, their interactions with aging and tumor immunity remain unexplained in MM.

Over the last decade, interest in the immune system's role in the onset and development of cancer has increased. The tumor microenvironment (TME) has generated considerable interest due to its composition of cancer cells and nonmalignant stromal cells, which involve various types of immune cells [11]. Tumor-infiltrating immune cells' activation status and structure are important criteria affecting tumor biology and diagnostic prediction. In many cancers, including MM, a high percentage of active CD8 T cells is associated with a longer patient survival period [12]. On the other hand, tumor-associated macrophages, mast cells, and neutrophil granulocytes all contribute to tumor progression, and their extensive invasion usually suggests a poor prognosis [12]. Cytotoxic CD8 T cells and CD4 helper T cells specifically target antigenic tumor cells to inhibit tumor growth [13].

Emerging immunotherapeutic methods, such as immunotherapy with anti CTLA4 [14] and anti-PD-1 antibodies, have shown some efficacy and enhanced patient survival [15]. However, 50–60% of patients undergoing these therapies do not undergo a prolonged reaction and have a bad prognosis [16, 17].

In our study, we developed an aging risk model to evaluate the immune microenvironment in malignant melanoma and to assess prognosis. A low aging risk was correlated with a low prognosis and suggested the presence of an immunosuppressive microenvironment. The aging signature is closely correlated with cellular and clinical features and can be seen as a predictive biomarker in patients with melanoma.

Subsequently, GSEA associated with low aging risk individuals were shown to be involved in carcinogenesis and immunosuppressive signaling. After that, we developed and validated an aging risk model that served as an independent prognostic indicator and accurately reflected the overall intensity of the immune response in MM.

## 2. Materials and Methods

### 2.1. Data Collection and Gene Acquisition

The RNA-seq transcriptome data and associated clinicopathological information for 471 melanoma patients were downloaded from the UCSC-accessible TCGA dataset (https://xenabrowser.net/datapages/) as a training set. Similarly, 214 melanoma patients were downloaded as a validation package from GEO as a validation set. The human aging genome resource (HAGR) identified 307 human aging genes (AGs) that were spread across all chromosomes.

### 2.2. Constitution of a Prognostic Risk Model and Efficacy Evaluation

To obtain the coefficients, aging genes that were statistically important in univariable Cox regression were then used in multivariable Cox regression; the risk-score function was constructed as follows:

(1) $$\begin{array}{c} \text{risk score}=\sum_{i=1}^{N}\left({\text{Exp}}_{i}\times {\text{Coe}}_{i}\right), \end{array}$$

where *N* = 3, Exp_*i*_ denoted the expression level of three aging-related genes, and Coe_*i*_ denoted the corresponding multivariable Cox regression coefficient.

### 2.3. Survival Analysis

Using the survival and survminer packages in *R*, a Kaplan–Meier analysis was used to compare overall survival (OS) between high and low aging risk groups. To distinguish potential prognostic factors, we used univariate Cox analysis and multivariate Cox analysis to define risk score as an independent risk factor for OS in melanoma. To check the risk model's precision in calculating the patients' OS, the survival ROC *R* package was used to build a ROC curve.

### 2.4. Gene Set Enrichment Analysis (GSEA)

GSEA was used to determine if there was a statistically important variation in the set of genes expressed between high aging and low aging risk classes in the MSigDB Collection enrichment. Each study included 1,000 permutations of the gene collection. A risk score was calculated using the phenotype mark.

### 2.5. The Development and Validation of the Immune Cell Type Fractions and Immune Checkpoint-Related Gene Signature

CIBERSORT is a computer program that uses gene expression data to estimate the relative abundances of member cell types in a mixed cell population [18]. LM22, a 547-gene leukocyte gene signature matrix, was used in CIBERSORT to identify 22 immune cell types, such as dendritic cell macrophages, macrophages M0, macrophages M1, macrophages M2, plasma cells, activated memory CD4 T cells, and CD8 T cells. They consist of 22 immune cell types in low and high aging risk scores and were calculated using CIBERSORT. The CIBERSORT output values were defined using the fraction of immune cells infiltrating a sample. In each case, the number of immune cell type fractions equaled one. Spearman rank correlation research was used to investigate the relationships between activity genes and the levels of infiltrating immune cells, and the results were visualized using the “ggplot2” software. The investigators looked at the association between the risk score and immune checkpoint gene expression levels (CTLA-4, PD-1, and PD-L1).To assess the novel gene signature's diagnostic ability in identifying MM patients, ROC analysis was performed on each identified gene in 471 patients with MM from the TCGA cohort and further validated in 214 MM patient samples from the GSE65904.

### 2.6. Verification of Expression Level and Prognostic Significance

To elucidate the translational level differential expression of three aging genes, the Human Protein Atlas (HPA) web database (http://www.proteinatlas.org/) was used to compare the expression of three aging-associated genes between normal and melanoma tissues.

## 3. Results

### 3.1. Characterization of Aging Risk Signature to Predict Melanoma

**Figure 1:**
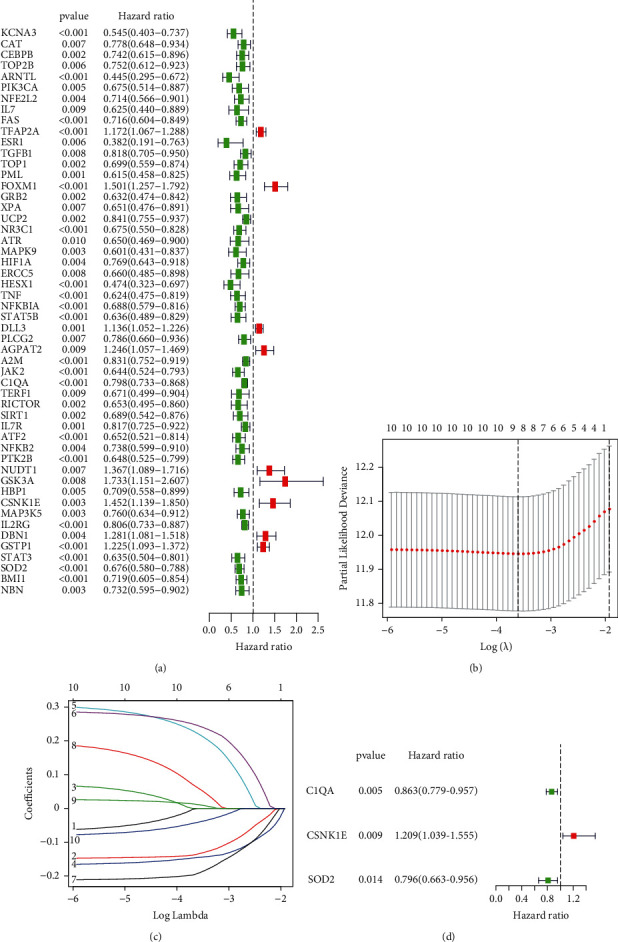
Construction of the aging-associated genes to predict prognosis of melanoma in the TCGA database. (a) Univariate cox regression analysis for the aim of identifying prognosis-associated aging genes using their HR, 95% CI, and *P* values from the training dataset. (b) Plots of the produced coefficient distributions for the logarithmic (lambda) series for parameter selection (lambda). (c) The LASSO analysis was used to identify the prognostic variables and develop the predictive models. (d) Multivariate cox regression identification of an aging risk signature for melanoma prognosis prediction.

309 aging-related gene sets were derived using GSEA. KCNA3, ARNTL, FAS, FOXM1, NR3C1, STAT5B, C1QA, SOD-2, GSK3A, and CSNK1E were among the 58 genes with the highest degree of involvement, suggesting that they are involved in melanoma (Figure 1(a)). The top 58 genes in the TCGA were used logarithmic (lambda) series for parameter selection and LASSO regression analyses to develop an aging risk signature for melanoma prognosis prediction (Figures 1(b) and 1(c)). CSNK1E, C1QA, and SOD-2 were chosen as the three aging-related genes with a *p* < 0.01 significance level in the multivariate Cox analysis to establish the predictive model (Figure 1(d)). The danger score was calculated by the formula:

(2) $$\begin{array}{c} \text{risk score}=\left(0.19\times \text{CSNK}1\text{E}\right)-\left(0.15\times \text{C}1\text{QA}\right)-\left(0.23\times \text{SOD}2\right). \end{array}$$

### 3.2. The Aging Risk Signature's Prognostic Value in Melanoma

**Figure 2:**
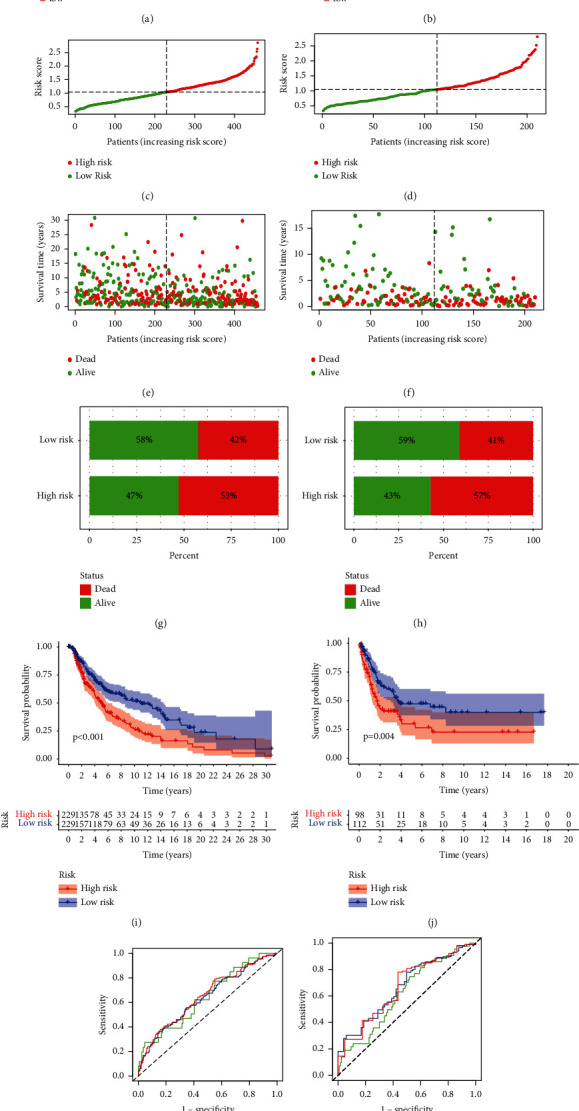
The aging risk signature's prognostic importance in malignant melanoma. A heatmap depicting the expression profiles of three aging genes in high aging and low aging risk model both in TGGA (a) and GSE65904 cohort (b). (c–f) Distribution of patient longevity status and risk score in high aging and low aging risk populations in the TCGA and GEO cohorts. The dot indicates the patient's status in ascending order of risk score. (g, h) Death rates in high aging and low aging risk groups for TCGA (i) and GEO (j) data survival curves. (k–l) Receiver operating characteristic curves for forecasting overall survival using TCGA and GEO datasets.

In both the TGGA and GEO datasets, as presented in the heatmap, increased expression of CSNK1E, an aging-related gene, was linked to higher risk scores, indicating that patients at significant risk develop an aging microenvironment. Additionally, in both datasets, SOD-2 and C1QA gene expression was decreased, which was correlated with low-risk levels (Figures 2(a) and 2(b)). According to our findings, the risk of death was marginally higher in the high aging risk population than in the low aging risk group (Figures 2(c)–2(h)).

Subsequently, the Kaplan–Meier procedure was used to determine the predictive value of an aging signature in melanoma. A high aging risk score was associated with a low OS rate in the TGGA (Figure 2(i)), and this association was confirmed in the GEO cohort (Figure 2(j)).

### 3.3. Aging-Related Genes in Melanoma Associated with Clinicopathological Characteristics

**Figure 3:**
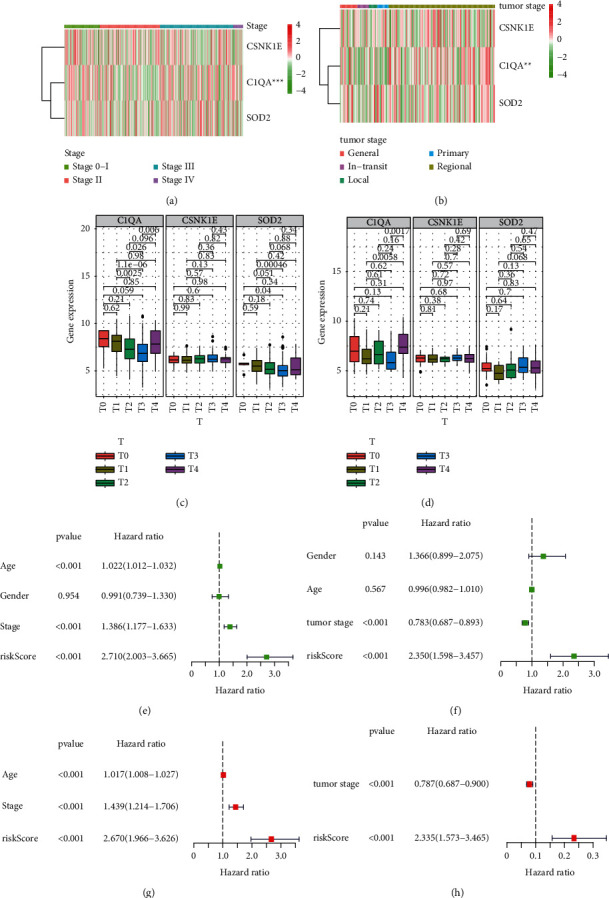
Expression of aging-associated genes with clinicopathological characteristics of melanoma. Three aging-associated genes' expression data at various tumor clinical stages in TGGA (a) and GEO datasets (b). Three AGs expression patterns in various tumor clinical stages of melanoma (c, d). Evaluation of the built prognostic model's efficacy. Cox regression study of the clinicopathological characteristics of the TCGA dataset with univariate (e) and multivariate (g) variables. Cox regression evaluation of the GSE65904 cohort's clinical and pathological properties for univariate (f) and multivariate (h) variables.

Given the critical biological roles of aging in tumorigenesis and development, we conducted a comprehensive study of the associations between three newly discovered aging-associated genes with melanoma clinical stages. Heatmaps show that the expression of CSNK1E, SOD-2, and C1QA was increased in different tumor clinical stages in the TCGA and GEO datasets (Figures 3(a)–3(d)).

Additionally, univariate and multivariate Cox regression analyses were used to determine if the risk signature was a significant independent predictor of outcome. In both the TCGA and GSE65904 databases, the risk score was a significant independent prognostic factor consistent with MM prognosis. The univariate analysis showed a significant association between a high aging risk score and short overall survival (OS) (Figures 3(e) and 3(f)). Additionally, variables such as age, grade, and stage were found to be predictive of poor survival. Multivariate analysis showed an independent correlation between a high aging risk score and a marginally shorter OS in patients with melanoma, implying that a high aging risk score may serve as a prognostic factor for melanoma. This was validated in the GEO database (Figures 3(g) and 3(h)).

### 3.4. Prognosis Evaluation Using Aging Risk Signatures

**Figure 4:**
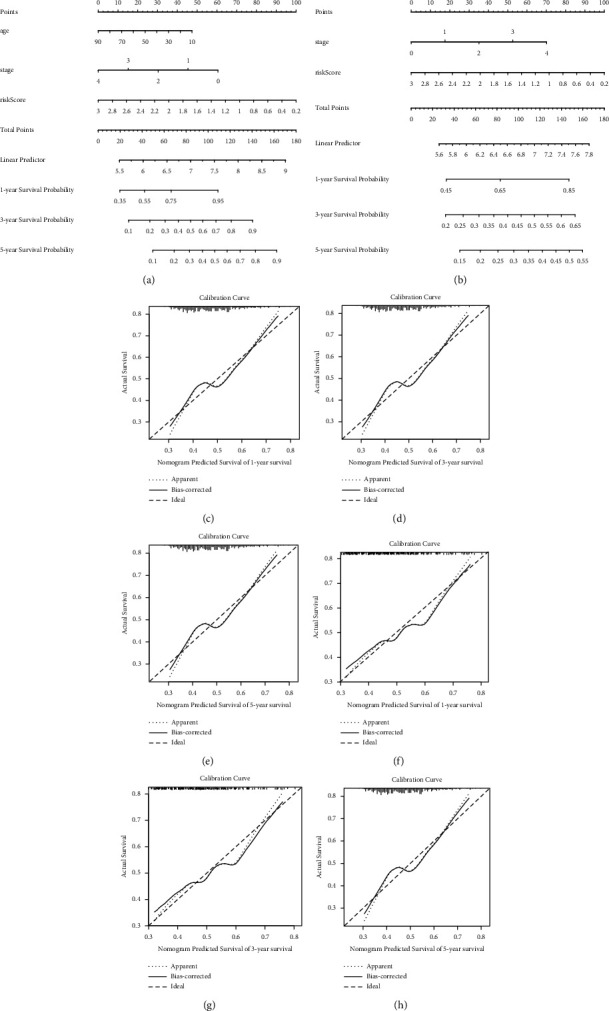
Construction of nomogram plots. (a-b) The risk score and other clinical conditions associated with nomograms were shown to predict overall survival time in the TCGA and GSE65904 cohorts. (c–e) The nomogram calibration plot in 1, 3, and 5 years in the TCGA dataset. (f–h) The nomogram calibration map to estimate 1-, 3-, and 5-year OS in the GSE65904 dataset.

The nomogram plot is another kind of statistical model that can be measured to forecast clinical outcomes in malignant melanoma. A nomogram plot was created using the risk score and other clinical features, allowing for the estimation of each patient's survival probabilities at one, three, and five years (Figures 4(a) and 4(b)). Calibration plots at three and five years showed excellent agreement between expected and reported results in both the TCGA and GSE65904 datasets (Figures 4(c)–4(h)). These results suggest that the prognostic model has a high predictive value for MM and clinical characteristics.

### 3.5. Gene Set Enrichment Analysis Categorizing Aging Signaling Pathways

**Figure 5:**
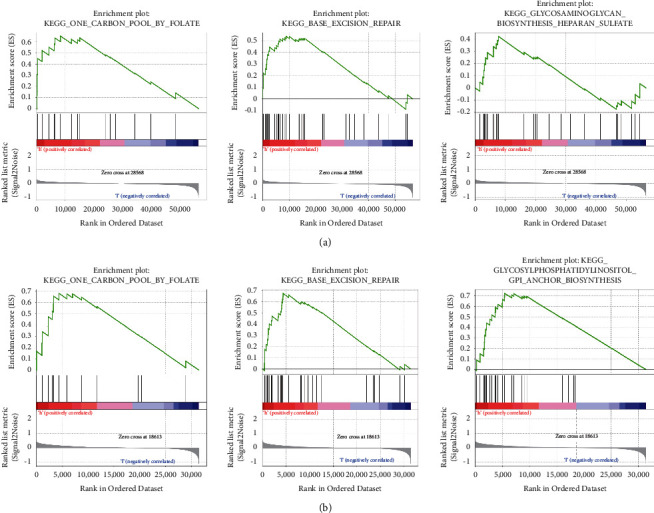
GSEA enrichment between different aging risk groups in TCGA and GSE65904 cohort. (a) GSEA revealed that genes associated with a higher risk of aging were enriched for malignant tumor hallmarks in the TCGA data: glycosylphosphatidylinositol (GPI)-anchor biosynthesis, base excision repair, and one carbon pool by folate. (b) The findings were further confirmed using GSE65904 data.

We used GSEA to assess high- and low-risk groups for aging to further substantiate the activation of similar signaling mechanisms in the high aging risk group. The TCGA database's high aging risk groups are differentially enriched for gene sets associated with processes such as glycosylphosphatidylinositol (GPI)-anchor biosynthesis, base excision repair, and one carbon pool by folate (Figure 5(a)). And then we validated in the GSE65904 dataset (Figure 5(b)).

### 3.6. Immune Cell Fractions in Different Risk of Aging in Melanoma

Numerous studies have shown that the senescent microenvironment may protect tumors from conventional antitumor immune responses by suppressing anticancer effector cells and stimulating immune escape. This study sought to determine if an aging vulnerability signature may be used to classify the immune microenvironment.

**Figure 6:**
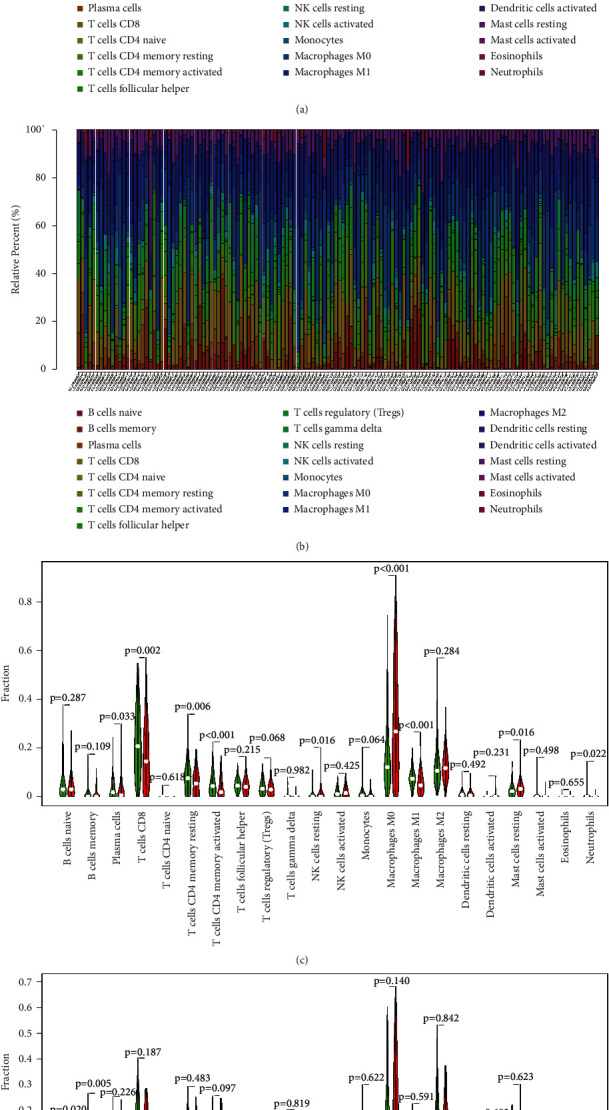
Consumption and description of immune cell penetration with low aging and a high aging risk in malignant melanoma. (a, b) Description of the approximate concentrations of twenty-two immune cells subtypes in TCGA dataset and GEO cohorts using the CIBERSORT algorithm. (c, d) Violin plot illustrating the association between the immune score and the low aging and high aging risk scores. The green and red colors denote samples with low aging and high aging risk samples, respectively.

We compared 22 immune cells in patients with low aging and high aging risk in melanoma using the newly developed CIBERSORT method. We visualized the findings in a box plot of differing shades of colour reflecting various immune cell subsets (Figures 6(a) and 6(b)). The findings from 471 melanoma patients in the TCGA and 214 patients in GSE65904 datasets

A violin plot illustrates the relationship between risk, resistance, and stromal scores. The red color represents the group with a high rate of aging, while the blue color represents the group with a low rate of aging. Different immune cell types were expressed differently in the high aging and low aging cohorts (Figures 6(c) and 6(d)).

### 3.7. Low-Risk Scores of Aging Implies an Immunosuppressive Microenvironment

**Figure 7:**
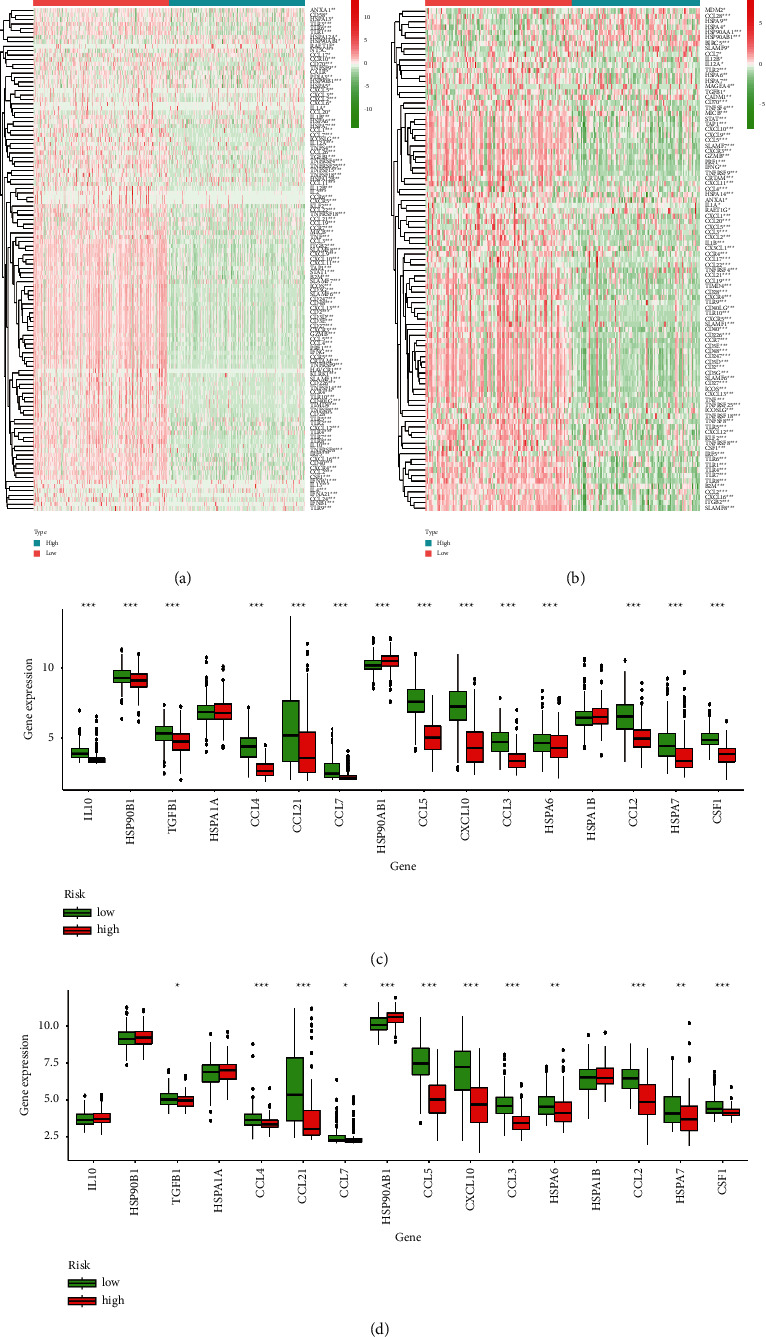
An immunosuppressive microenvironment is shown by a low aging risk ranking. Heatmap of the gene profiles involved in the negative regulation of the cancer-immunity cycle in high and low aging risk classes, respectively, from the TCGA (a) and GEO datasets (b). Regulation of tumor immunosuppressive cytokines in high aging and low aging risk populations foraging in the (c) TCGA and (d) GEO datasets.

The expression of genes that exist as negative regulators of these processes was examined in individuals at low and high risk of aging. Gene signatures are obtained in the database Tracking Tumor Immunophenotype [19]. AGs involved in negative control were mostly upregulated in the low aging risk group, suggesting that these patients have a low level of process activation. We verified the expression of immunosuppressive cytokines in MM with a low aging and a high aging risk, based on previous indications that these molecules are upregulated in reaction to low-level stimuli (Figures 7(c) and 7(d)).

**Figure 8:**
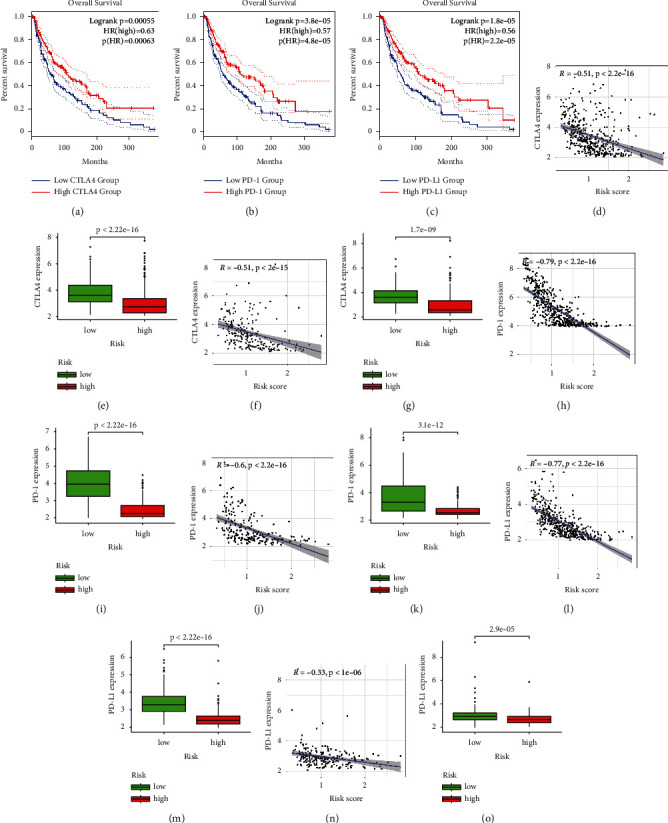
A low aging risk score shows an immunosuppressive microenvironment. (a–c) In the TCGA dataset, Kaplan–Meier analysis demonstrated the prognostic value of CTLA-4, PD-1, and PD-L1 in melanoma. (d–o) CTLA-4, PD-L1, and PD-1 expression in TCGA and GEO cohorts with high and low aging risk.

As seen in Figures 8(a)–8(c), the overall survival was shorter in MM patients with lower immune checkpoints in the TCGA cohort. Our findings indicated that the expression of essential immune checkpoints (CTLA-4, PD1, and PD-L1) was upregulated in the low aging risk population, which was negatively associated with the aging risk score. Immunosuppressive cytokines were also increased in the population with a low risk of aging (Figures 8(d)–8(o)). These findings suggest that patients with low aging risk scores are more likely to experience an immunosuppressive microenvironment as a result of increased expression of immunosuppressive cytokines and immune checkpoints.

### 3.8. Validation of Aging-Associated Gene Expression and Evaluation of Clinicopathological Features in MM

**Figure 9:**
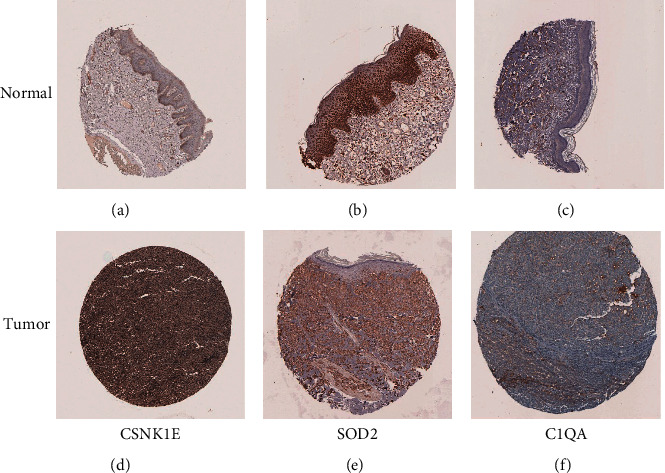
The HPA database validates three AGs proteins correlated in melanoma and normal skin tissue. (a, d) CSNK1E, (b, e) SOD-2, and (c, f) C1QA.

To further characterize their expression in MM, we used immunohistochemistry evidence from the HPA (Human Protein Atlas) database to demonstrate that CSNK1E was substantially increased in melanoma compared to normal skin tissue (Figures 9(a)–9(d)). However, when melanoma tissue was linked to normal skin tissue, the amount of antibody staining for SOD-2 and C1QA was significantly lower as shown in Figures 9(b), 9(c), 9(e), and 9(f).

## 4. Discussion

Cancer and aging are intrinsically related, resulting in a perplexing and uneasy pathologic union [20]. The processes behind this interaction have absorbed researchers around the world, but are largely based on the cancer cell itself. However, far less research has been focused on aging in the microenvironment.

One of cancer's primary features is the cells' ability to resist immune suppression [21]. In the 1960s, research established a connection between aging and decreased immune function. Subsequent research has proven that a chronically inflammatory microenvironment promotes aging [4]. The most noticeable age-related transition believed to affect the progression and aggressiveness of the cancer is a decline in the immune system's effectiveness over time. With aging, inflammation and asthenic immune surveillance can promote the formation and development of tumors [21]. Melanoma, or the malignant transformation of epidermal melanocytes, is the primary cause of death from skin cancer globally. Age is a significant prognostic factor, and elderly melanoma patients have a lower disease-specific survival rate, although primary tumor causes are controlled. A previous study indicated that fibroblasts in the aged dermal microenvironment (age >55 years) promote melanoma tumor development by secreting factors that facilitate metastasis and resistance to targeted therapy. While age-related changes in tumor molecular pathways and the host immune response may account for some of these findings (2), little attention has been paid to the effect of age on the architectural changes that may control immune and tumor cell trafficking through the skin. Therefore, it is critical to investigate the expression patterns of AGs to comprehend the function of the aging process in MM.

This study examined the associations between 58 differentially expressed AGs and MM prognosis (Figures 1(a) and 1(b)) and constructed a prognosis risk model using three AGs, namely, CNK1E, SOD-2, and C1QA (Figure 1(c)). Furthermore, it revealed a robust performance signature for prognosis prediction compared to clinicopathological factors in training and multiple validations (Figure 3). SOD-2 and C1QA acted as protective factors in the prognostic risk model, while CSNK1E acted as a risk factor (Figure 4).

CSNK1E, a clock gene that controls circadian rhythms, has been shown to suppress tumor cell development selectively. CSNK1E was shown to induce cell cycle arrest at the G1 phase, thus mediating antitumor effects in HNSCC when upregulated by MM [22]. High levels of CSNK1E expression have been associated with a poor prognosis (i.e., shorter overall survival) in patients with ovarian cancer [23], but have been associated with a favorable prognosis in subsets of patients with breast cancer [24]. In MM, elevated CSNK1E expression was associated with a poor prognosis irrespective of other clinical conditions [25]. CSNK1E was previously identified as a possible candidate for the development of high-therapeutic-index anticancer agents. These findings support the theory that circadian clock genes can regulate cell cycle and survival signaling and emphasize the critical role of CK1 and PERIOD2 in connecting these processes. Tiong et al. found that CSNK1E-P53 could be the independent prognosis markers from stage for CRC patient survival [26]. Our findings are consistent with those of their study, indicating that SOD-2 and C1QA can act as anticancer genes. SOD-2 is a key cellular antioxidant enzyme that plays a critical role in regulating oxidative stress by catalyzing the conversion of superoxide to hydrogen peroxide [27, 28]. SOD-2 as either a tumor suppressor or promoter is intimately linked to its function as a regulator of mitochondrial oxidants [28].

Overexpression of SOD-2 has been shown to prevent prostate cancer cell proliferation, invasion, and growth [27]. Li confirmed that upregulation of SOD-2 in breast cancers was suggested to function as a tumor suppressor gene [29]. As a result, the function of SOD-2 in tumorigenesis has been and continues to be extensively studied. The preceding suggests that loss of SOD-2 expression could be a tumor-initiating phenotype and that SOD-2 as tumor suppressor function is primarily related to its role as an O2 scavenger throughout tumorigenesis [28]. Ross established a six-gene prostate cancer prognostic signature [30] and discovered that increased C1QA expression was correlated with improved overall survival. C1QA encodes the A-chain polypeptide of Complement C1q, which is a critical component of the TME [31], implying that C1QA could also be involved in melanoma. The primary producers of C1QA are macrophages, monocyte-derived dendritic cells, and some cell lines such as THP1. C1QA, a protein that produces amyloid and forms amyloid plaques, has been implicated in the pathogenesis of AD, one of the main aging-related diseases [32]. Additionally, Yang identifies PTAFR and C1QA as prognostic immune-related genes in the cutaneous melanoma tumor microenvironment as the most closely linked genes in the PPI network [33]. C1QA can function as a tumor promoter by facilitating cancer cell adhesion, migration, and proliferation, as well as angiogenesis and metastasis. C1QA is produced in the tumor microenvironment and functions as an extracellular matrix protein, promoting tumor growth and metastasis. These results show that, regardless of C1QA activation, C1QA will lead to tumor progression and invasion [31].

The immune system plays a crucial role in detecting and eradicating tumor cells [34]. During aging, the tumor microenvironment can affect the outcome of age-related immune dysfunction [35]. For example, with age, thymic activity decreases, leading to a decrease in the proportion of naive T cell subsets and a rise in the proportion of memory T cell populations, thereby impairing T cells' ability to mount responses to novel tumor-associated antigens [36] Adaptive immune responses, including cellular (T cells) and humoral (B cells), are the primary tumor control mediators [37]. Stimulating T cells and natural killer cells against tumor antigens is a critical event in antitumor immune surveillance. Antigens must be delivered to T cells on human leukocyte antigen (HLA) molecules by triggering an adaptive immune response. In terms of tumor surveillance and elimination, APCs will provide them with cytotoxic T (CD8+) lymphocytes via HLA I Helper T (CD4+) cells which are also involved and are activated through the interaction of HLA II molecules with APCs. Once activated, helper T cells release cytokines that enhance the function of B cells and cytotoxic T cells. Memory T cells and regulatory T cells (CD4+, CD25+, and FoxP3) are produced. Regulatory T cells are necessary to avoid collateral tissue damage when the immune system is not under control. Cutaneous immune cells recognize and process malignant changes such as melanoma and nonmelanoma skin malignancies through these processes that present and engage costimulatory molecules [38, 39].

Immune checkpoints are expressed on many immune cells such as T cells, regulatory B cells (Bregs), dendritic cells (DCs), natural killer cells (NKs), regulatory T (Tregs), M2-type macrophages, and myeloid-derived suppressor cells (MDSCs) [38]. Immune checkpoints contribute to carcinogenesis by improving tumors' immunosuppressive ability. To prevent immunological damage, immune inhibitory molecules such as CTLA-4 [37], PD-1, PD-L1 [32], and LAG-3 [40] block immune responses by negatively modulating immune cell signaling pathways. Our study's low aging risk group represented immune checkpoints CTLA-4, PD-1, and PD-L1. Consistent with this data, our study found that patients at high risk of aging had increased M0 macrophages and CD8 T cells but a decrease in activated CD4 T cells, suggesting an immune disability status in this population.

Macrophages are classified as M1 or M2 macrophages. M1 macrophages generate nitric oxide synthase, interleukin-12 (IL-12), and tumor necrosis factor-alpha (TNF-*α*), whereas M2 macrophages produce IL-10, TGF-*β*, and prostaglandin E2 [41–43]. It is well established that M1 macrophages contribute to the anticancer response [44]. It is well established that M1-like macrophages contribute to the anticancer response, while M2 macrophages promote angiogenesis and tissue remodeling, which contribute to tumor formation and immunosuppression1 [44]. CIBERSORT showed a slight increase in the proportion of M2 macrophages in our cohort of patients at high risk of aging. Immunosuppressive cells such as Treg cells and neutrophils were also increased in the elderly. Our aging risk model might be capable of forecasting the immune microenvironment. Cytokines play a key role in tumor immune regulation. Cancer-associated immunosuppressive cytokines are a significant contributor to immune cell fatigue. TGF-*β* has been shown to suppress the immune system in advanced malignancies by impairing NK cell activity, decreasing cytokine production, impairing dendritic cell maturation, and modifying the cytotoxic properties of T cells [45]. Interleukin-10 (IL-10), a potent immunosuppressive cytokine produced by M2-macrophages, Tregs, and Th2-cells, has been shown to inhibit effector T cell proliferation, cytokine production, and migration [46]. In our study, immunosuppressive cytokines were upregulated in communities at low risk of aging. The tumor microenvironment has a significant impact on cancer cell fate determination. The combination of stimulatory and inhibitory signals can alter the direction of antitumor immune responses to tumor antigens.

## 5. Conclusion

We formulated an aging risk model focused on three aging-associated genes (C1QA, CSNK1E, and SOD-2). This risk model reflects the immune microenvironment and the efficacy of immunotherapy in MM patients. We now have a clear insight into how aging influences the immune microenvironment of melanoma, which may be used as a prognostic indicator that helps the creation of prospective melanoma therapies.

## Data Availability

The data used to support the findings of this study are obtained from publicly accessible datasets.
